# Pinnacle^3^ modeling and end‐to‐end dosimetric testing of a Versa HD linear accelerator with the Agility head and flattening filter‐free modes

**DOI:** 10.1120/jacmp.v17i1.5808

**Published:** 2016-01-08

**Authors:** Daniel L. Saenz, Ganesh Narayanasamy, Wilbert Cruz, Nikos Papanikolaou, Sotirios Stathakis

**Affiliations:** ^1^ Department of Radiation Oncology University of Texas Health Science Center—San Antonio San Antonio TX USA

**Keywords:** beam modeling, end‐to‐end testing, treatment planning, quality assurance

## Abstract

The Elekta Versa HD incorporates a variety of upgrades to the line of Elekta linear accelerators, primarily including the Agility head and flattening filter‐free (FFF) photon beam delivery. The completely distinct dosimetric output of the head from its predecessors, combined with the FFF beams, requires a new investigation of modeling in treatment planning systems. A model was created in Pinnacle^3^ v9.8 with the commissioned beam data. A phantom consisting of several plastic water and Styrofoam slabs was scanned and imported into Pinnacle^3^, where beams of different field sizes, source‐to‐surface distances (SSDs), wedges, and gantry angles were devised. Beams included all of the available photon energies (6, 10, 18, 6 FFF, and 10 FFF MV), as well as the four electron energies commissioned for clinical use (6, 9, 12, and 15 MeV). The plans were verified at calculation points by measurement with a calibrated ionization chamber. Homogeneous and heterogeneous point‐dose measurements agreed within 2% relative to maximum dose for all photon and electron beams. AP photon open field measurements along the central axis at 100 cm SSD passed within 1%. In addition, IMRT testing was also performed with three standard plans (step and shoot IMRT, as well as a small‐ and large‐field VMAT plan). The IMRT plans were delivered on the Delta^4^ IMRT QA phantom, for which a gamma passing rate was >99.5% for all plans with a 3% dose deviation, 3 mm distance‐to‐agreement, and 10% dose threshold. The IMRT QA results for the first 23 patients yielded gamma passing rates of 97.4%±2.3%. Such testing ensures confidence in the ability of Pinnacle^3^ to model photon and electron beams with the Agility head.

PACS numbers: 87.55.D, 87.56.bd

## I. INTRODUCTION

The Elekta Versa HD Agility head introduces a number of novel components which could uniquely impact beam modeling. A dynamic leaf guide with variable thickness out‐of‐field blocking, no backup jaws, and flattening filter‐free (FFF) capabilities are included. One hundred and sixty 5 mm (projected width at isocenter) multileaf collimators (MLCs) travel up to 3 cm/s over the full $40\times 40\;{\text{cm}}^{2}$ field‐of‐view.^(1)^ The MLC carriage can travel at 3.5 cm/s for a maximum MLC leaf speed of 6.5 cm/s. FFF beam commissioning requires new analytic source models,^(2)^ and the physical properties of such beams have been modeled with Monte Carlo.^3^, ^4^, ^5^

The mean energy in an FFF beam is reduced, resulting in shallower depth of maximum dose ($d_{\text{max}}$) and reduced TPR 20/10 values.^6^, ^7^ The decreased mean energy for the FFF photon beams is a consequence of less beam hardening.^(8)^ In addition, removal of the flattening filter in the FFF beams leads to different electron contamination spectra.^(9)^ FFF beams can lower the out‐of‐field dose, another parameter directly affecting beam modeling.^(10)^ Furthermore, the Versa HD possesses unique FFF beam profiles due to the MLC‐defined collimation, possibly leading to larger transmission penumbra through the rounded shape of MLCs. The widened penumbra, combined with the sharp FFF profiles, further complicates the full width half maximum (FWHM) of FFF profiles. Multileaf collimation closer to the source dictates an alternate head leakage spectrum and higher geometric penumbra, and leads to tighter tolerance for positional accuracy.^(11)^

Flattening filter‐free beams are not new to radiotherapy and are implemented in a variety of linear accelerators in addition to the Elekta Versa HD, including the Varian TrueBeam (High Intensity Mode), and Siemens (high‐intensity unflat beams), as are described in the recent AAPM Therapy Emerging Technology Assessment Work Group report on flattening filter‐free accelerators.^(12)^ The beam modeling of FFF beams has been successfully commissioned in a variety of treatment planning systems previously, including Pinnacle^3^ collapsed cone convolution superposition, as well as Eclipse Acurus XB/analytical anisotropic algorithm and CMS XiO, among others.^13^, ^14^ These studies were limited to Varian linear accelerators, however (TrueBeam and 23EX). Other studies have investigated FFF beams of an Elekta Precise.^(15)^ The Elekta Versa HD high dose rate FFF mode uses an independent energy set for the FFF beams compared to the conventional flattened beams, unlike the Varian approach which uses the identical electron beam for both modes. The independence of the Elekta FFF mode suggests that there exists less of a clear relationship between the spectra between FFF and flattened beams. Furthermore, although the reduction in electron contamination can be modeled, the Versa HD and TrueBeam differ in the composition of the material which replaces the flattening filter (stainless steel versus brass), which may lead to distinct electron contamination. These previous findings indicate that, while the modeling of an FFF beam is readily achievable, the implementation of the Agility‐specific FFF may carry specific adaptations from the aforementioned studies.

With such a broad array of advancements between Versa HD and its predecessors impacting the workflow from beam modeling through treatment delivery, the results of an end‐to‐end dosimetric test can also ensure confidence in the ability to calculate the delivered dose. Moreover, such testing is recommended after treatment planning system commissioning by AAPM TG‐40 and after treatment planning system changes by AAPM TG‐142, as well as TG‐53.^16^, ^17^, ^18^ While VMAT and arbitrary field‐specific measurements have been previously reported with the Agility collimator,^(19)^ a complete postcommissioning end‐to‐end dosimetric test has not been published. This study carries out this work for the Versa HD linear accelerator for photon modalities with various geometries and for electrons in a simple geometry, as specified in TG‐53.

## II. MATERIALS AND METHODS

### A. Beam modeling

All measurements were performed with the PTW MP3‐M water phantom (PTW, Freiburg, Germany). Commissioned beam data at 100 cm SSD included percent depth‐dose (PDD) curves and in‐plane and cross‐plane profiles for square field sizes from $1\times 1\;{\text{cm}}^{2}$ to $40\times 40\;{\text{cm}}^{2}$. Output and wedge factors were acquired at a reference depth of 10 cm with a PTW Diode P dosimeter $\left(\text{sensitive volume}=0.03\;{\text{mm}}^{3}\right)$ for field sizes $<5\times 5\;{\text{cm}}^{2}$ and with a PTW Semiflex 31010 Chamber (sensitive volume 0.125 cc) for fields $\geq 5\times 5\;{\text{cm}}^{2}$. Similarly for PDDs, the Diode P and Semiflex chamber were utilized for fields smaller and larger than $5\times 5\;{\text{cm}}^{2}$, respectively. The Diode P was used for profile scans of all field sizes. Output factors were acquired with the PTW Semiflex 31010 Chamber for fields $5\times 5\;{\text{cm}}^{2}$ to $40\times 40\;{\text{cm}}^{2}$, while the PTW Diode P was used for smaller field sizes down to $1\times 1\;{\text{cm}}^{2}$. A daisy chaining approach was used for the small field output factors using the overlapping $5\times 5\;{\text{cm}}^{2}$ reading.

Electron data was acquired with the Semiflex chamber for cone factors as well as PDDs and profiles. Data were smoothed with a weighted three‐point algorithm with PTW MEDPHYSTO mc^2^ software version 3.2 and symmetrized before import into Pinnacle^3^ v9.8 (Philips Radiation Oncology Systems, Fitchburg, WI) for beam modeling. Model profiles and percent depth‐dose data were calculated, followed by a global automodeling optimization sequence. This sequence conducts a course spectrum tuning first at $10\times 10\;{\text{cm}}^{2}$ followed by other field sizes and then expands to cross beam high‐dose and low‐dose regions and optimizes the spectral off‐axis softening. Electron contamination is then optimized again starting at $10\times 10\;{\text{cm}}^{2}$ and proceeding in a similar fashion with focus on the buildup region. The sequence also separates the X and Y components of focal spot size tuning, as well as jaw/MLC transmission. Following automodeling, no further adjustments were made to the spectrum. For out‐of‐field parameters, the effective source size required manual refinement for wedged fields to obtain sharp shoulders at the edges of beam profiles. This was accomplished with an effective source size smaller than that resulting from automodeling. MLC transmission was set as needed, based on measurement and MLC‐defined field penumbra agreement.

The Pinnacle^3^ dose calculation engine is based on the convolution algorithm developed by Mackie and Papanikolaou, creating a beam model projecting the energy fluence as it exits the treatment head throughout the patient and superimposing a Monte Carlo calculated kernel describing the dose deposition.^20^, ^21^, ^22^ A collapsed cone convolution superposition (CCCS) model depositing dose along discreet cone central axes is employed for speed.^(23)^ Heterogeneities are accounted for in the primary fluence and scatter dose by considering the radiological depth and density scaling the kernels. Numerous studies have validated the high accuracy of CCCS, particularly in its ability to model dose in regions of radiation disequilibrium such as in the buildup region at tissue interfaces.^24^, ^25^, ^26^, ^27^ For the purposes of this study, the “Adaptive Convolve” and “Electron 3D” algorithms were utilized for dose calculation for photons and electrons, respectively.

Electron beam modeling proceeded relatively simply, compared to photons, since the electron implementation was fairly conventional in the Versa HD. Spatial dose distributions come from Fermi‐Eyges multiple scattering theory with small angle scattering. The lateral distribution in Pinnacle^3^ is described as a Gaussian function with the FMCS (water scatter correction factor) parameter controlling the magnitude of the angular spread. Sigma‐theta‐x (angular scattering variance) was used to account for changes in penumbra size with depth. One aspect requiring specific attention was the electron cone size, being defined at 95 cm from the source instead of 100 cm. When inputting electron field sizes in the electron physics module, the projected cone sizes at 100 cm were entered instead of the nominal cone size (e.g., $10\times 10\;{\text{cm}}^{2}$ cone was entered as $10.5\times 10.5\;{\text{cm}}^{2}$, reflecting the field size at isocenter).

### B. Verification measurements

**Figure 1:**
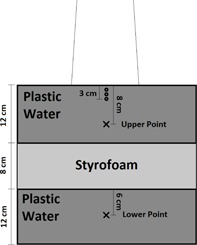
Phantom used for end‐to‐end testing point dose measurements. An “X” indicates a photon measurement point (the upper point at 8 cm depth is for homogeneous phantom measurements and the lower at 26 cm depth is for heterogeneity testing). An “O” indicates an electron measurement point (1 cm depth for 6 MeV, 2 cm for 9 MeV, and 3 cm for 12 and 15 MeV).

Following beam modeling in Pinnacle^3^, verification measurements were made. A plastic water (CNMC, Nashville, TN) phantom (overall dimensions of $30\times 30\times 32\;{\text{cm}}^{2}$) with an 8 cm slab of Styrofoam centered in the middle was scanned on a GE Discovery LightSpeed CT scanner (GE, Milwaukee, WI) and exported to Pinnacle^3^ for treatment planning. The phantom is outlined in Fig. 1. Presence of streaking artifacts near the edge of the phantom resulted in density being overridden to 1.04 g/cm^3^, as specified in the manufacturer's guidelines. For all of the photon energies, the following beams were devised. Beam 1 consisted of an AP (${\text{gantry angle}=0}^{\circ }$, per IEC 1217 specification) with a $10\times 10\;{\text{cm}}^{2}$ open field with the phantom surface at 100 cm SSD. Beam 2 added a 30° universal wedged field to beam 1. The 30° wedged field is a combination of an open field and a wedged field with the fixed universal 60° wedge in place in the gantry head. The use of a 30° wedged field tests not only the wedged beam model, but also the combination of a wedged and open field for an effectively smaller angle wedged field. Beam 3 utilized an extended SSD of 110 cm and a field size of $20\times 20\;{\text{cm}}^{2}$ to test the output factor, as well. Beam 4 rotated the gantry to 20° off the central axis with a field size of $30\times 30\;{\text{cm}}^{2}$. The measurement point was 2.5 cm off of the central axis for the oblique beam. Beam 5 consisted of a rectangular field of 20×5 cm with jaws defining the 20 cm edge. For the flattening filter‐free beams, beam 2 with the wedge was not included. Heterogeneity tests included only beam 1 (open field) and beam 4 (oblique incidence) to test different amounts of overlying heterogeneities. For the electrons, two fields were devised. Beam 1 was an open field with a $10\times 10\;{\text{cm}}^{2}$ cone. Beam 2 utilized an extended SSD (105 cm). 200 MU was prescribed for all photon and electron beams. Table 1 summarizes the beam parameters.

Two points of interest (POIs) were added in the Pinnacle^3^ plan representing the location of the ionization chamber. The upper point was placed at 8 cm and the lower point at 26 cm depth along the central axis. The lower point was 6 cm behind the Styrofoam slab for heterogeneity testing. For electrons, the point of the chamber was placed at 1 cm for 6 MeV, 2 cm for 9 MeV, and 3 cm for the 12 and 15 MeV plans. The dose at the upper and lower POI due to each beam was tabulated. The fields were then sent to the record‐and‐verify system, MOSAIQ (Elekta, Stockholm, Sweden), for delivery at the treatment vault.

**Table 1:** Summary of fields used for end‐to‐end dosimetric verification.

				*Photons*			
*Beam*	*Description*	*Gantry Angle*	*Wedge*	*SSD*	*Field Size*	*Applicable Energies*	*Points Measured*
1	Open Field	0	None	100 cm	$10\times 10\;{\text{cm}}^{2}$	All	Upper, Lower
2	30° wedge	0	Universal	100 cm	$10\times 10\;{\text{cm}}^{2}$	Flattened	Upper, Lower
3	110 cm SSD	0	None	110 cm	$20\times 20\;{\text{cm}}^{2}$	All	Upper
4	Oblique	20	None	100 cm	$30\times 30\;{\text{cm}}^{2}$	All	Upper, Lower
5	Rectangular	0	None	100 cm	$20\times 5\;{\text{cm}}^{2}$	All	Upper, Lower
				*Electrons*			
*Beam*	*Description*	*Gantry Angle*	*Wedge*	*SSD*	*Cone Size*	*Applicable Energies*	*Points Measured*
1	Open Field	0	N/A	100 cm	$10\times 10\;{\text{cm}}^{2}$	All	Energy dependent
2	105 cm SSD	0	N/A	105 cm	$10\times 10\;{\text{cm}}^{2}$	All	Energy dependent

Point‐dose measurements were made using the previously described phantom. A calibrated PTW 31013 Semiflex 0.3 cc ionization chamber was positioned in a plastic water slab with the sensitive volume of the chamber centered in the slab and along the beam's central axis. The TG‐51 protocol was followed for dosimetric measurements. Values for $P_{\text{ion}}$ and $P_{\text{pol}}$ were measured during TG‐51 calibration as a part of commissioning. A PTW UNIDOS webline electrometer was used for charge collection with a bias of −300 V.

### C. IMRT end‐to‐end testing

IMRT plans, already under investigation in advanced applications such as FFF for SBRT and SRS radiosurgery,^28^, ^29^ were subject to end‐to‐end dosimetric testing with flattened‐beam plans delivered to a biplanar array of diode dosimeters, Delta^4^ (ScandiDos, Uppsala, Sweden). The array consisting of 1069 diodes with 5 and 10 mm spacing in the center and periphery, respectively, was commissioned for the VersaHD linac as specified in the manufacturer's guideline. Passing criteria for gamma analysis was 90% of all points with gamma below 1 using a 3% dose difference and 3 mm distance‐to‐agreement criteria. A 10% dose threshold was used, and the normalization level was specified at 90% of the maximum measured dose (an approximation of the prescription dose given the presence of hot spots). The dose normalization is, therefore, not done with respect to a normalization point, but instead to a normalization dose level. In this manner, 3% dose deviation is relative to the prescription dose. VMAT plans with small and large fields ($5\times 4\;{\text{cm}}^{2}$ and $21\times 12\;{\text{cm}}^{2}$ at isocenter, respectively) were chosen, along with a step‐and‐shoot IMRT plan. Measurements were conducted on three occasions on different days. In addition, IMRT QA results from the first 23 patient treatment plans were analyzed.

## III. RESULTS

### A. Beam modeling

**Figure 2:**
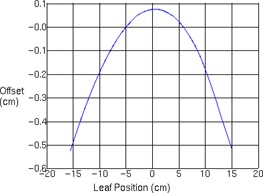
Offset of the dosimetric leaf edge used for dose calculation compared to the physical position of the leaf end projected to isocenter.

**Figure 3:**
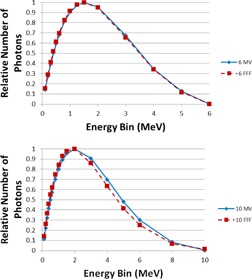
Energy spectra for 6 and 10 MV flattened and FFF beams.

MLC leaf transmission was modeled as 0.00479 and 0.00328 for 6 MV and 6 FFF, respectively. For 10 MV and 10 FFF, leaf transmission was set to 0.00356 and 0.00163 respectively. The interleaf leakage through 9 cm thick tungsten MLCs was measured in another study at 0.4% for 6 MV and 0.5% for 10 MV.^(1)^ Such low MLC leakage is novel for IMRT, for which accurate leaf transmission data are critical.^(30)^ 18 MV was modeled with a leaf transmission of 0.00163. Leaves were specified as tungsten of 9 cm thickness, with a maximum of 3 cm/s travel speed. Figure 2 shows the modeled rounded leaf shape in Pinnacle^3^ describing the offset of the dosimetric leaf edge used for dose calculation compared to the physical position of the leaf end projected to isocenter. Photon spectra are shown for 6 and 10 MV flattened and FFF beams in Fig. 3 and indicate reduced relative numbers of photons in the high‐energy bins for FFF due to less beam hardening in the flattening filter. Flattening filter attenuation was modeled as an arbitrary profile rather than a cone, as has been the case for other FFF beams in the literature.^(31)^ Wedged fields were modeled with a separate model. For the flattened fields, large field wedged profiles exhibited a shoulder near the penumbra which was eliminated by editing the arbitrary profile fluence for this wedge model. Final dose computation was performed with 3 mm resolution after inspection of small field profiles at 1 mm resolution. Open field and wedged output factors are tabulated in 2, 3. 4, 6 show the measured and modeled PDDs, open profiles, and wedged profiles for a variety of field sizes. An MLC‐only defined $10\times 10\;{\text{cm}}^{2}$ field (lower jaws open out of the way with a 20 cm field width) is also presented in Fig. 7. Table 4 summarizes the modeled parameters for each photon energy. The spectral off‐axis softening parameter (SOASP) describes the change in the photon spectrum for rays directed at an angle off of the central axis due to transmission through differing amounts of the flattening filter. The reduction in each spectral bin i is modified by the factor

(1) $${\left[1/(1+E_{i/E_{\text{max}}})\right]}^{SOASP\times \Theta }$$

where *Θ* is the off‐axis angle, $E_{i}$ is the relative number of photons in the ith energy bin, and $E_{\text{max}}$ is the maximum photon energy in the photon spectrum.^(32)^

**Table 2:** Output factors (Sc,p) measured at 10 cm depth in water (100 cm SSD) for all photon energies and field sizes $1\times 1\;{\text{cm}}^{2}$ through $40\times 40\;{\text{cm}}^{2}$. For field sizes 1×1 through $4\times 4\;{\text{cm}}^{2}$, the daisy‐chaining calculation with respect to the $5\times 5\;{\text{cm}}^{2}$ is shown. The calculation is represented as $\left(\text{output factor from Diode})\times (\text{output factor from chamber at}\;5\times {5\;\text{cm}}^{2}\right)$.

*Square Field (cm)*	*6 MV*	*10 MV*	*18 MV*	*6 FFF*	*10 FFF*
1	0.761×0.906	0.705×0.923	0.665×0.935	0.764×0.927	0.739×0.945
	0.690	0.650	0.622	0.709	0.698
2	0.889×0.906	0.879×0.923	0.853×0.935	0.896×0.927	0.898×0.945
	0.806	0.811	0.798	0.830	0.848
3	0.931×0.906	0.936×0.923	0.930×0.935	0.942×0.927	0.950×0.945
	0.844	0.863	0.870	0.873	0.898
4	0.972×0.906	0.972×0.923	0.970×0.935	0.975×0.927	0.984×0.945
	0.880	0.897	0.907	0.904	0.930
5	0.906	0.923	0.935	0.927	0.945
7	0.951	0.959	0.968	0.962	0.972
8	0.970	0.975	0.981	0.977	0.983
10	1.000	1.000	1.000	1.000	1.000
12	1.026	1.022	1.018	1.018	1.012
15	1.058	1.047	1.040	1.040	1.026
20	1.096	1.076	1.062	1.062	1.041
25	1.122	1.094	1.075	1.076	1.050
30	1.141	1.108	1.088	1.086	1.056
35	1.153	1.117	1.094	1.091	1.059
40	1.159	1.120	1.095	1.093	1.060

**Table 3:** Relative wedge factors for the universal wedge for the three flattened photon beams measured at 10 cm depth in water (100 cm SSD).

*Square Field (cm)*	*6 MV*	*10 MV*	*18 MV*
5	0.220	0.238	0.228
10	0.251	0.263	0.253
15	0.269	0.281	0.270
20	0.293	0.290	0.283
30	0.302	0.313	0.296
34	0.306	0.313	0.294

**Figure 4:**
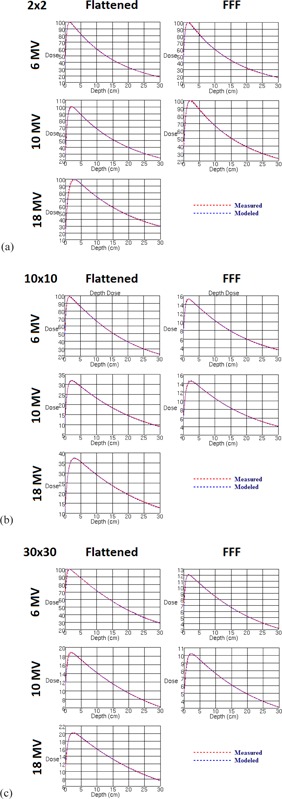
Measured vs. modeled percent depth‐dose curves for all photon energies (a) 2 cm, (b)10 cm, and (c) 30 cm square fields. Measurements were made with a PTW 31010 Semiflex 0.125 cc ionization chamber.

**Figure 5:**
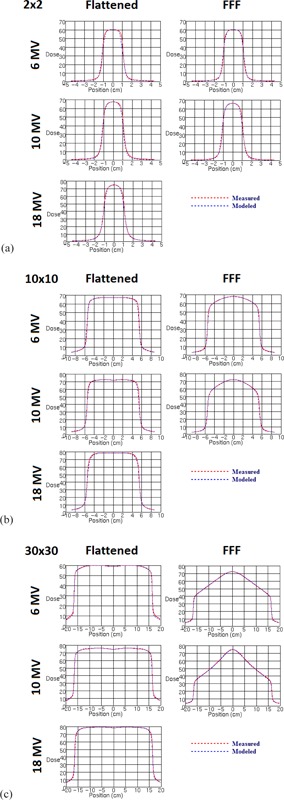
Measured vs. modeled profiles along the inline jaw‐defining axis for all photon energies (a) 2 cm, (b) 10 cm, and (c) 30 cm square fields. Measurements were made with a PTW Diode P detector.

**Figure 6:**
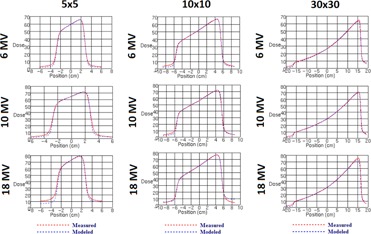
Measured vs. modeled wedged profiles for all flattened beam energies measured at 10 cm depth (5 cm, 10 cm, and 30 cm square fields).

**Figure 7:**
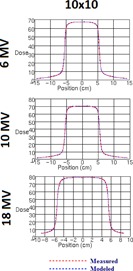
Measured vs. modeled profiles of MLC‐only defined $10\times 10\;{\text{cm}}^{2}$ field measured at 10 cm depth in water at 100 cm SSD.

**Table 4:** Summary of Pinnacle^3^ modeled photon beam parameters by energy.

Spectral off‐axis softening factor (open)	12.22	10.97	19.84	‐1.19	1.5
Spectral off‐axis softening factor (wedged)	3.41			N/A	N/A
Effective Source Size (open) (cm)	0.12×0.12	0.12×0.14	0.07×0.14	0.14×0.14	0.09×0.14
Effective Source Size (wedged) (cm)	0.16×0.30			N/A	N/A
Jaw Transmission (open)	0.002	0.002	0.0036	0.003	0.002
Jaw Transmission (wedged)	0.001			N/A	N/A
MLC Leaf Transmission (open)	0.00479	0.00356	0.00 163	0.00328	0.00163
MLC Leaf Transmission (wedged)	0.03038			N/A	N/A
Maximum MLC Leaf Speed			3 cm/s		
MLC Leaf Thickness			9 cm		

### B. Verification measurements

5, 6 show the results of the end‐to‐end Plastic Water phantom validation with an 8 cm Styrofoam slab along the central axis. Percent difference between locally measured and calculated dose relative to overall maximum dose is presented in 5, 6 for the upper point (with no heterogeneities) and lower point (heterogeneity evaluation point), respectively. Each row corresponds to the distinct beam arrangements and modifications used (including $10\times 10\;{\text{cm}}^{2}$ open field, a wedged field, extended SSD, oblique incidence, and a rectangular $20\times 5\;{\text{cm}}^{2}$ field). Good agreement is observed within 1% for the open and rectangular field measurements. Similarly, all FFF beam measurements were within 1%. Low readings for the oblique fields were noticed for the flattened beam energies (‐1%). Finally, the output with a wedge surpassed 1% for 10 and 18 MV beams only.

At the lower point, beneath 12 cm of plastic water, 8 cm of Styrofoam, and another 6 cm of plastic water, open field heterogeneity tests were all within 2%. For oblique incidence, 1% agreement was observed. Results did not vary systematically across energy.

Electron results are illustrated in Table 7. The upper homogeneous portion of the Plastic Water phantom was used for electron measurements. The two measurement geometries used were a $10\times 10\;{\text{cm}}^{2}$ open field at 100 cm SSD as well as an extended SSD of 105 cm. Agreement was within 1% for 6 MeV and within 2% for all other electron energies at both 100 cm SSD and 105 cm SSD.

**Table 5:** Upper point (8 cm depth) percent differences between measured and planned point doses for different beam parameters and for each photon energy in the Plastic Water phantom.

	*6 X*	*10 X*	*18 X*	*6 FFF*	*10 FFF*	*Average Absolute % Difference*
Open Field	−0.5%	0.1%	−0.2%	0.3%	0.3%	0.3%
30° wedge	0.5%	1.1%	1.1%	N/A	N/A	0.9%
110 cm SSD	−1.0%	−0.4%	−0.7%	−0.1%	0.2%	0.5%
Oblique	−1.0%	−1.0%	−1.0%	0.5%	−0.4%	0.8%
Rectangular	−0.2%	−0.1%	0.0%	0.1%	0.4%	0.2%

**Table 6:** Lower point (26 cm depth) percent differences between measured and planned point doses for different beam parameters and for each photon energy in the heterogeneity phantom.

	*6 X*	*10 X*	*18 X*	*6 FFF*	*10 FFF*	*Average Absolute % Difference*
Open Field	0.9%	1.5%	1.4%	1.4%	1.4%	1.3%
30° wedge	1.2%	1.8%	1.5%	N/A	N/A	1.5%
Oblique	0.8%	0.9%	1.0%	0.8%	0.7%	0.8%
Rectangular	0.8%	1.3%	1.4%	1.1%	1.2%	1.2%

**Table 7:** Percent difference between measured and planned point doses for different beam parameters and for each electron energy in the Plastic Water phantom.

	*6 MeV*	*9 MeV*	*12 MeV*	*15 MeV*	*Average Absolute % Difference*
Open Field	0.8%	1.3%	1.4%	1.4%	1.2%
105 cm SSD	0.5%	1.2%	1.7%	1.5%	1.2%

### C. IMRT end‐to‐end testing

**Figure 8:**
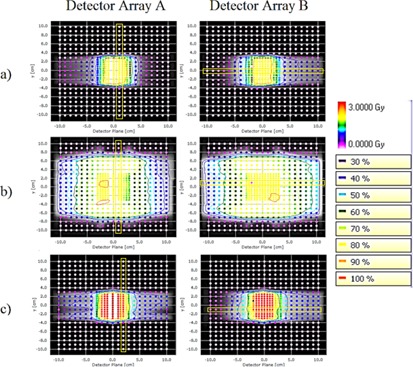
Isodose lines from IMRT QA measurements for (a) a small‐field VMAT plan, (b) a large‐field VMAT plan, and (c) step‐and‐shoot IMRT. The background brightness levels indicate the calculated dose distribution, while the overlayed points show the dose measurements. Yellow boxes indicate the location of profiles shown in Fig. 9.

**Figure 9:**
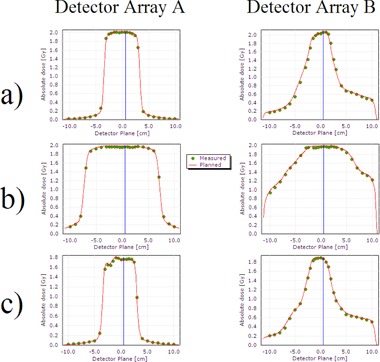
Dose profiles resulting from IMRT QA measurements for (a) a small‐field VMAT plan, (b) a large‐field VMAT plan, and (c) step‐and‐shoot IMRT. Points indicate measurements, while the solid curve is the calculated dose distribution. The profile locations are indicated in Fig. 8.

Table 8 outlines the passing rates for the standard IMRT QA plans measured with Delta^4^ for a small field VMAT arc ($5\times 4\;{\text{cm}}^{2}$), a large field VMAT arc ($21\times 12\;{\text{cm}}^{2}$), and a step‐and‐shoot IMRT plan. Measurements were repeated three times. The gamma passing rate was above 99.5% for all three IMRT QA plans using 3% dose deviation, 3 mm distance to agreement criteria. Figure 8 shows the two‐dimensional dose distribution with isodose lines, while line profiles of the planned versus the measured dose distributions are shown in Fig. 9. Agreement extends beyond the field edges to the low‐dose regions, as well as along the high‐dose and high‐dose gradient regions. Table 9 presents the same data analyzed with passing criteria of 2%/2 mm. For the 23 IMRT QAs of the first clinically treated patient plans, we measured passing rates of 81.2%±12.5% for dose deviation, 97.7%±1.6% for distance to agreement, and 97.4%±2.3% for overall gamma.

**Table 8:** Results of the gamma analysis for IMRT QA measurements for a variety of IMRT configurations (passing criteria of 3%/3 mm).

	Passing Rate (%)		
	<3% *Dose Deviation*	<3 mm *DTA*	*Gamma*
Small Field Arc	71.1%±3.1%	98.2%±1.2%	99.8%±0.0%
Large Field Arc	95.8%±0.5%	99.5%±0.5%	99.6%±0.2%
Step & Shoot	77.2%±3.1%	99.6%±0.0%	99.8%±0.0%

**Table 9:** Results of the gamma analysis for IMRT QA measurements for a variety of IMRT configurations (passing criteria of 2%/2 mm).

	*Passing Rate (%)*		
	<2% *Dose Deviation*	<2 mm *DTA*	*Gamma*
Small Field Arc	58.5%±3.9%	82.9%±4.1%	91.7%±2.5%
Large Field Arc	80.0%±1.5%	87.4%±0.9%	94.9%±0.9%
Step & Shoot	63.8%±0.7%	95.1%±0.6%	95.9%±1.1%

## IV. DISCUSSION

The end‐to‐end testing results indicate agreement between the treatment planning system and ionization chamber measurements to within 2%, matching at a minimum the ICRU recommendation for a low‐dose gradient region.^(33)^ As a point of reference, Cunningham^(34)^ suggested that beam calibration accuracy can be within 2.5%, as part of the overall principle of a total of 5% accuracy in radiotherapy. For a more rigorous assessment, a comparison of the results with the expected agreement thresholds in TG‐53 Table 4‐4 was performed in order to determine the appropriateness of the results. The thresholds are based on region discrimination (e.g., normalization point, central axis, in‐field, out‐of‐field) in phantom based on the work of Van Dyk et al.^(35)^ For the open field measurements on the central axis, TG‐53 reported an expected agreement of 1%, which is met for both the FFF and flattened photon beams. For a wedged field, the expected agreement is within 2%, which is also met for all photon beams. For the 110 cm SSD variation, TG‐53 expected 1% agreement along the central axis. In this case, only the measured data for 6X extended SSD beam is right on threshold at 1.0%, which was confirmed with multiple measurements. The other energies passed within 1% for extended SSD. For the oblique and rectangular fields, the agreements are both within TG‐53 expectation of 1.5%. Table 4‐4 in TG‐53 specifies agreement within 3% for heterogeneous slabs, which was satisfied in this study for all energies. The particularly deep 26 cm total depth with a large 8 cm heterogeneity leads to an extreme measurement beyond typical patient geometry where a 3% tolerance may be inappropriate. Nevertheless, agreement was within 1% for 6 MeV and 2% for other energies. Electron percent depth‐dose curves are characterized by their sharp dose gradient beyond the depth of maximum dose. Therefore, one would expect difficulties in precisely verifying a point dose at a depth just beyond $D_{\text{max}}$. For energies 9, 12, and 15 MeV, the measurement point was just deeper than $d_{\text{max}}$ (2 cm versus 1.8 cm for 9 MeV, 3 cm versus 2.4 cm for 12 MeV, and 3 cm versus 2.6 cm for 15 MeV). In those cases, agreement was larger than 1% but less than 2%. For 6 MeV on the other hand, the point was just shallower than $d_{\text{max}}$ (at 1 cm versus 1.2 cm), where agreement was more easily achieved within 1%. While other factors may be involved, sharp dose gradients may be responsible in part for the nevertheless small deviation.

Good agreement in IMRT QA passing results indicated no major inaccuracies in beam modeling, such as those which could have resulted from improper specification of MLC leaf transmission or leaf travel speed. Treatment delivery of VMAT plans was rapid compared to step‐and‐shoot IMRT. This rapid nature of VMAT is a partial result of the leaf speed (as well as other factors, including reduced total MU for VMAT plans and gantry speed), which was modeled at a maximum speed of 3 cm/s, verified partially by the lack of beam‐on delays due to leaves not being in their proper position. Dose deviation pass rates were generally lower than the distance‐to‐agreement criteria, typical for our institutional experience with Delta^4^.

## V. CONCLUSIONS

End‐to‐end dosimetric testing ensured the accuracy of the Versa HD beam models in Pinnacle^3^ v9.8. Homogeneous and heterogeneous measurements were within the tolerances summarized above based on the expectations of AAPM Task Group 53. The tests ensure dosimetric, as well as mechanical, accuracy through the testing of absolute dose calibration and beam modeling parameters. Efficient VMAT plans were deliverable with the Agility head with no compromise in delivered dose distribution accuracy.
